# Book Notices

**Published:** 1894-11

**Authors:** 


### Book Notices.

A MANUAL OF THERAPEUTICS. By A. A. Stevens, A. M., M. D.. Lecturer on Terminology and Instructor in Physical Diagnosis in the University of Pennsylvania; Demonstrator of Pathology in the Woman's Medical College, Philadelphia; Physician to St. Mary's Hospital and to the Southeastern Dispensary; Pathologist to St. Agnes's Hospital. In one volume of 435 pages. Price, in cloth, $2.25. W. B. Saunders, publisher, 925 Walnut street, Philadelphia. 1894.

As its name indicates, this book is only a manual, and is intended especially for students. The author has endeavored to furnish the beginner in the study of therapeutics with an "outline of modern therapeutics, to be filled in and extended by systematic study of the larger works." The advantages to the student of first becoming familiar with the classification of drugs, together with their physiological action and small range of therapeutical application, before attempting to master a knowledge of their many and varied uses as taught in the larger works, is obvious to any one who has passed over this stage. The great difficulty with the beginner is that he finds more in his text book on therapeutics than he is able to master, and repeated attempts often result in confusion. It is to give him a clear idea of the ground work upon which to build his superstructure that Dr. Stevens has prepared this excellent manual.

The book is the result of careful study; it is concise and practical and is superior to the average manual, aud we heartily commend it to all medical students. H.

---

CLINICAL DIAGNOSIS. By Albert Abrams, M. D. (Heidelberg), Professor of Pathology, Cooper Medical College, San Francisco, Cal.; Pathologist to the City and County Hospital, San Francisco; Author of "A Synopsis of Morbid Renal Secretions," etc.; President of the San Francisco Medico-Surgical Society (1893–'94); President of the Alumai Association of Cooper Medical College (1888–'89). Third edition, revised and enlarged. In one octavo volume of 273 pages, with 28 illustrations and handsomely bound. Price, cloth $2.75. E. B. Treat, publisher, 5 Cooper Union, New York.

The first edition of Dr. Abrams' Clinical Diagnosis was issued in 1890, and it has met with so favorable a reception at the hands of the medical profession that in the short space of four years it has passed to the third edition. This work is not so voluminous as many others, but owing to the excellent arrangement and the many synoptic tables, it is one of the most valuable ready reference works on this subject, In this edition a chapter on insanity, a summary of recent methods of diagnosis and many synoptic tables have been added. This last addition will prove of much value to the student and practitioner, and greatly enhances the value of the volume. We heartily commend the book to the profession. H.

---

THE POCKET ANATOMIST. By C. Henry Leonard, A. M., M. D., Professor of Gynecology of Detroit College of Medicine. Leather, 300 pages, 193 illustrations, postpaid $1.00. The Illustrated Medical Journal Co., Detroit, Mich.

The 18th edition of this popular anatomy is now before us; it is printed upon thin paper and bound in flexible leather so as to be specially handy for the pocket. The illustrations are photo-engraved from the English edition of Gray's Anatomy, so are exact as to their details. Three large editions have been sold in England, testifying to the popularity there, and some sixteen thousand copies have been sold in this country. It briefly describes each artery, vein, nerve, muscle and bone, besides the several special organs of the body. It contains more illustrations than any of the other small anatomies.

---

ESSENTIALS OF PHYSICS.—Arranged in the form of questions and answers. Prepared especially for students of medicine. By Fred J. Brockway, M. D., Assistant Demonstrator of Anatomy at the College of Physicians and Surgeons, New York. Second edition, revised, 339 pages, 155 illustrations. Price, cloth, $1.00 net. Interleaved for notes, $1.25 net. W. B. Saunders, Publisher, 925 Walnut St., Philadelphia.

The necessity for a book on medical physics, one more complete than the elementary works on this subject, and not so large as to require too much time in mastering it, is very apparent. It is a deplorable fact that a very large number of medical students are totally ignorant of physics when they matriculate, and it is for the purpose of furnishing these students with a work on this subject, giving them all the necessary information and in the shortest possible form, that this book has been written.

The author claims no originality, but he has succeeded in producing a most excellent compilation. The subjects could hardly be presented in a more concise or clearer way. The book will doubtless become very popular with medical students, and it deserves to be. H.

---

THE DISCOVERY OF MODERN ANÆSTHESIA--BY WHOM WAS IT MADE?. A Brief Statement of Facts. By Dr. Laird W. Nevius, Specialist in the administration of Nitrous Oxide Gas for Minor Surgery and the Painless Extraction of Teeth, Cooper Institute, New York. 111 pages. Printed on fine heavy paper, with a number of full page portraits. Price, cloth, $1.00.

This is the most complete history of the discovery of modern anæsthesia that we have ever seen. The author has endeavored to give an impartial and unprejudiced statement of the claims of the four contestants—Dr. Crawford W. Long, Dr. Horace Wells, Dr. W. T. G. Morton, and Dr. Charles T Jackson for priority in the discovery. He does not claim to have discovered anything new bearing on the subject, but has compiled facts and figures, showing when and where the four noted men were born, where they received their professional education, when and where they performed their first operations using an anæsthetic, etc.

After giving a careful history of all the circumstances attending the first uses of ether, nitrous oxide gas and chloroform to produce anæsthesia for surgical work, the author makes the following statements:

1. That Dr. Crawford W. Long, of Athens, Georgia, discovered that sulphuric ether was an anæsthetic and that under its influence surgical operations could be and were performed by him in the year 1842.

2. That Dr. Horace Wells, of Hartford, Conn., discovered that nitrous oxide gas was an anæsthetic and that under its influence surgical operations could be and were performed by him in the year 1844.

3. That Dr. William T. G. Morton, of Boston, Mass., first publicly demonstrated to the world that capital surgical operations could be and were performed without pain under the influence of sulphuric ether, in the year 1846.

4. That Dr. James Y. Simpson, of Edinburgh, Scotland, discovered that prolonged surgical operations could be and were performed by him without pain under the use of chloroform, in the year 1847. H.

---

TREATMENT OF TYPHOID FEVER. By D. D. Stewart, M. D., Lecturer on Clinical Medicine in the Jefferson Medical College, of Philadelphia; Physician to the Medical Dispensary of the Episcopal Hospital; formerly Attending physician to St. Mary's Hospital, and to St. Christopher's Hospital for Children; Fellow of the College of Physicians of Philadelphia. One hundred and four pages. Price, in paper covers, 25 cents; cloth, 50 cents. George S. Davis, Publisher, Detroit, Mich.

The opening chapter is on the "Prophylaxis of Typhoid Fever, General and Special," in which the source of infection and the pathogenic germ are discussed.

The second chapter is devoted to the "General Management of a Case of Typhoid Fever." In this chapter, the author gives minute instruction for the care and nursing of the patient, including the diet, the care and ventilation of his room, the bath, the disinfection of the feces, etc.

In the third chapter, the subject of "Specific and Antiseptic Treatment of Typhoid Fever" is discussed. The author gives the preference to the Brand method when it is practicable, but when it is not (and this is the case with the majority of patients we meet in private practice), he favors the antiseptic treatment, and believes that beta-naphthol is the best of all the antiseptic agents that have yet been proposed.

The fourth, and last, chapter is devoted to the consideration of the "Treatment of Special Symptoms and complications." In this chapter, the many complications that come up during the course of the disease are carefully considered, and specific rules are laid down for their management. This chapter is a very important one, owing to the fact that it deals with the symptoms that give the physician the greatest difficulty in the management of typhoid fever cases. The book is an excellent one, and will assist the physician much in his work. H.

---

GONORRHŒA: Being the translation of Blenorrhœa of the Sexual Organs and its complications. By Dr. Ernest Finger, Docent at the University of Vienna. One volume, of 330 pages, octavo, illustrated by numerous wood engravings, and by seven chromo-lithographic plates. Third revised and enlarged edition. Bound in muslin, gold lettered, $3.00. William Wood & Company, New York.

There is, perhaps, no disease that the physician is more frequently called on to treat than gonorrhœa, and certainly none that occasions him more worry and anxiety, and in which he meets with more disappointments. Until very recent years the treatment of this disease was entirely empirical and routine, and only since the advent of bacteriology has some light been thrown on its pathology, and a rational line of treatment become possible. The discovery of the gonococcus, the possibility of its more ready culture and more precision in the question of mixed infections, are important advances made in recent years. One of the most important contributions to the literature of this subject is Dr. Finger's excellent monograph, of which this is the third edition. Many valuable additions have been made in the present edition, and it constitutes an exhaustive treatise, fully abreast of the times, containing all the recent bacteriological investigations as well as the most approved methods of treatment. H.

---

TRANSACTIONS OF THE SOUTHERN SURGICAL AND GYNECOLOGICAL ASSOCIATION, volume VI. Sixth session held at New Orleans, La., November 14, 15 and 16, 1893. Published by the Association, 1894.

This volume, like its predecessors, is a credit to the Association and to the publishing committee. It makes a handsome book of nearly four hundred octavo pages, and is well filled with excellent articles on surgical and gynecological subjects. The papers presented at this meeting of the Association cover a wide range of subjects, and were contributed by men eminent in the medical profession. They are valuable contributions to the literature of surgery and gynecology, and their publication in substantial book form will be appreciated by the entire profession. A volume of transactions so attractive in appearance, and containing so much matter worthy of preservation, merits much praise. H.

